# Data in support of metabolic reprogramming in transformed mouse cortical astrocytes: A proteomic study

**DOI:** 10.1016/j.dib.2014.09.004

**Published:** 2014-11-07

**Authors:** Azeddine Bentaib, Pascal De Tullio, Hervé Chneiweiss, Emmanuel Hermans, Marie-Pierre Junier, Pierre Leprince

**Affiliations:** a GIGA-Neuroscience, University of Liège, Liège, Belgium; b Pharmaceutical Chemistry, Department of Pharmacy, University of Liège, Liège, Belgium; c Glial Plasticity and Cerebral Tumors, UMR8246 CNRS/U1130 Inserm/ UMCR18, Université Pierre et Marie Curie, Paris, France; d Institute of Neurosciences, Group of Neuropharmacology, Université Catholique de Louvain, Brussels, Belgium

## Abstract

2D-DIGE analysis coupled with mass spectrometry is a global, without a priori, comparative proteomic approach particularly suited to identify and quantify enzymes isoforms and structural proteins, thus making it an efficient tool for the characterization of the changes in cell phenotypes that occur in physiological and pathological conditions. In this data article in support of the research article entitled “Metabolic reprogramming in transformed mouse cortical astrocytes: a proteomic study” [1] we illustrate the changes in protein profile that occur during the metabolic reprogramming undergone by cultured mouse astrocytes in a model of in-vitro cancerous transformation [2].

## Specifications table

**Table:** 

Subject area	Biology

More specific subject area	Cancer biology, Neurobiology
Type of data	Table, figures
How data was acquired	Fluorescence scanning of bi-dimensional electrophoretic separations of pre-labeled protein samples, using a Typhoon 9400 scanner.
	Mass spectrometry of peptides generated from proteins in 2D gel plugs, using an UltraFlex II spectrometer (Brüker Daltonics).
Data format	Analyzed using DeCyder 7.0 program
Experimental factors	Proteins extracted from treated and control cultures in a detergent and urea containing buffer, then labeled with CyDyes.
Experimental features	2D electrophoresis (1st dimension pH 3–10 gradient; 2nd dimension 12.5% PAGE). Spot picking with EtanDalt Spotpicker. Trypsin digestion of proteins and MALDI-TOF/TOF analysis of the peptides.
Data source location	N/A
Data accessibility	Data is provided in Supplementary materials directly with this article.

## Value of the data

• Quantitative proteomics study exploring the changes in cellular protein abundance that result from metabolic reprogramming following astrocyte transformation.
• Multiple changes occur with similar amplitude for enzymes catalyzing successive steps in canonical metabolic pathways.
• Illustration of the power of 2D-DIGE to reveal (and quantify) changes in abundance of multiple isoforms of enzymes and cellular proteins.

## 1 Data, experimental design, materials and methods

Using the 2D-DIGE approach we have obtained relevant information on the changes in abundance of most enzymes implicated in major canonical metabolic pathways [1]. Supplementary Table 1 lists all the identified proteins whose abundance differs by at least 1.5-fold (*p*<0.05, student *t*-test) between normal astrocytes (NA) and transformed astrocytes (TA).

Our results suggest that transformation causes major losses of astrocyte-specific proteins and functions and the acquisition of metabolic adaptations that favor intermediate metabolites production for increased macromolecule biosynthesis. We also observe a loss of some enzymes implicated in the oxidative stress defense. This is illustrated in Supplementary materials showing zoomed-in 2D gels regions where the spots containing the proteins/enzymes that underwent these changes (e.g. Pyruvate kinase, Lactate dehydrogenase, Glutamate dehydrogenase, Glutamine synthetase, Transaldolase, Peroxiredoxin 1 and 6, Glutathione S-transferase Mu1…) are visible, together with a graphical representation of the quantitative data.

### 1.1 Cell culture preparation

Cultures of mouse normal astrocytes were prepared from cortices of 1-to-2-day-old C57Bl6/J mice according to protocols described by Sharif in 2007 [3] and Prevot in 2005 [4] with slight adaptations. Briefly, cultures were established in MEM medium (Gibco, Life-technologies, UK) containing 10% fetal calf serum (FCS) (Lonza, BioWhittaker^®^, Belgium). Medium was changed every 2 days following washes with ice-cold phosphate-buffered saline (PBS, pH 7.4). When confluence was reached, cultures were shaken overnight (250 rpm), trypsinized and seeded in 8 cm dishes at a density of 50,000 cell/cm^2^. When confluence was reached again, the cells were maintained in serum-free MEM medium for 48 h, rinsed twice in PBS, and either extracted for protein sample preparation or frozen until further use. Glial fibrillary acidic protein (GFAP)-immunoreactive astrocytes accounted for 91–96% of the cells in the NA cultures, the remaining 4–9% of cells being CD11b receptor-immunoreactive microglia [3].

The transformed astrocytes are obtained from normal mouse astrocytes that had undergone a treatment with TGF alpha allowing them to dedifferentiate into “neural progenitor-like cells” followed by gamma irradiation causing their in vitro transformation [2]. Transformed astrocytes are cultured in medium composed of a mixture of DMEM and F12 medium (Invitrogen, France), containing 0.6% glucose, 2 mM glutamine, 13 mM sodium bicarbonate, 5 mM N-2-hydroxyethylpiperazine-N׳-2-ethanesulfonic acid (HEPES) buffer, 5 IU/ml penicillin and 5 µg/ml streptomycin, and the B27 complement (Gibco, ref 12587-010). This medium was renewed once a week. Due to the selection process and growth conditions, all the cells generated from the irradiated cultures were transformed astrocytes.

### 1.2 Sample preparation for comparative proteomic studies

NA and TA were collected in lysis buffer (7 M urea, 2 M thiourea, 30 mM Tris, pH 8.5 (all from GE Healthcare) and 2% (w/v) ASB-14 (Sigma)) and sonicated for 10 min. After removal of the insoluble material by centrifugation (20.000*g*), each sample was precipitated using the 2D Clean-up kit (GE Healthcare) in order to remove salts and low molecular weight contaminants that interfere with CyDye-labeling and protein electrophoresis. Protein pellets were resuspended in adequate volume of lysis buffer and the pH was adjusted to 8.5 with 100 mM NaOH for efficient CyDye labeling.

Protein concentration was evaluated with the RC/DC Protein Assay (Bio-Rad Laboratories).

### 1.3 2D-DIGE

2D-DIGE comparative proteomic analysis was performed using a set of 4 independent cultures in each experimental condition (NA and TA).

For analytical gels, 25 µg of each biological replicate were labeled separately with 200 pmol of CyDye (Cy3, Cy5; GE Healthcare) with dye-swapping to minimize dye-specific labeling effect, vortexed, and incubated 30 min in the dark. At the same time, a pool of equal amounts of proteins from all the biological replicates was labeled with Cy2 for use as an internal standard. After 30 min in the dark, the reaction was stopped with 10 mM lysine. Multiplexing was achieved by pooling 25 µg of Cy3- and Cy5-labeled samples together with 25 µg of Cy2-labeled internal standard, used for matching and normalization between gels, and the volume of the combined labeled samples was adjusted to 450 µL with standard rehydration buffer (7 M urea, 2 M thiourea, 2% w/v ASB 14, 25 mM DTT, and 0.6% v/v pH 3–10 NL IPG buffer (GE Healthcare)). The mixed CyDye-labeled samples were used to rehydrate 24 cm IPG strips (pH 3–10 NL, GE Healthcare) for 12 h at 20 °C. Isoelectric focusing (IEF) was carried out at 500 V for 1 h, followed by two successive 3 h gradients (1st gradient, 500–1000 V, 2nd gradient 1000–8000 V), and constant setting at 8000 V for 70 kVh at 20 °C. The maximum current setting was 50 mA per strip in an IPGphor isoelectric focusing unit (GE Healthcare).

Prior to second dimension separation, the IPG strips were incubated 15 min at room temperature (RT) in equilibration buffer (30% glycerol (v/v), 1.6% SDS (w/v), 6 M urea, 50 mM Tris–HCl pH 8.8) containing 1% DTT and then for 15 min in the same solution containing 5% iodoacetamide. They were then sealed with 0.5% agarose in SDS running buffer, and placed on top of 12.5% w/v polyacrylamide gels. The second dimension electrophoresis was performed overnight at 30 °C in an Ettan Dalt-6 system (GE Healthcare) at a 50 V constant voltage. Each gel was finally scanned with the Typhoon 9400 scanner (GE Healthcare) at the emission wavelengths corresponding to each CyDye, namely 520 nm (Cy2), 580 nm (Cy3) and 670 nm (Cy5).

### 1.4 Image analysis

Images (*n*=4 for each experimental condition) were analyzed with the DeCyder 7.0 software (GE Healthcare) according to the manufacturer׳s instruction. In brief, co-detection and quantification of the three CyDye-labeled forms of each spot was performed using the Differential In-gel Analysis (DIA) module, allowing the calculation of ratios between samples and internal standard abundances for each spot. Matching of spots across all gels and correction of inter-gel variability through normalization of the Cy2 internal standard spot maps present in each gel was realized by the Biological Variance Analysis (BVA) module. Protein spots that showed a statistically significant Student׳s *t*-test (*p*<0.05) for an average spot abundance change of at least 1.5 were subjected to identification by MALDI-TOF/TOF mass spectrometry.

### 1.5 In-gel digestion and mass spectrometry (MS) analysis

Preparative gels that contained 200–300 µg of either NA or TA protein samples and 25 µg of the internal standard were run in parallel and matched to the analytical gels master image to generate picking lists of differentially expressed spots used to drive automated gel plug collection by the Ettan Spotpicker (GE Healthcare). The proteins in gel pieces were subsequently in gel digested as described by Mathy and colleagues [5], according to Shevchenko and colleagues protocol [6]. The gel pieces were sequentially washed 3 times with 25 mM NH_4_HCO_3_ and 100% acetonitrile (ACN) to remove excess of detergent and buffer. After the last dehydration in ACN, piece of gels were rehydrated for 1 h at 4 °C with 2 µL of 5 µg/mL trypsin proteomic grade solution (Roche) diluted in 25 mM NH_4_HCO_3_ in order to ensure sufficient trypsin diffusion and to prevent autocatalysis. Finally the temperature was increased to 37 °C for an overnight digestion.

Peptides were extracted by adding 5 μL of a 1% trifluoroacetic acid (TFA) (v/v)/30% ACN (v/v) solution and vortexing for 30 min. 2 μL of the resulting extract were deposited on a 384–600 MTP Anchorchip MALDI target plate (Brüker Daltonic) previously spotted with a 30 mg/mL (w/v) of α-cyano-4-hydroxycinnamic acid (CHCA) matrix solution (Sigma) diluted in acetone. After drop drying, each spot was desalted with cold 10 mM ammonium phosphate solution. Samples were analyzed with an UltraFlex II MALDI-TOF/TOF (Brüker Daltonics) by MS fingerprint (spectra acquisition mass range: 70–4240*m*/*z*). Peaks with the highest intensities obtained in TOF-MS mode were subsequently analyzed by LIFT MS/MS (mass range 40–1100). Proteins identifications were carried out with the biotools software (Brüker) using the Mascot search engine. The generated peak lists were used for mascot searching (www.matrixscience.com) using the following parameters for both PMF and MS/MS experiments: trypsin digestion, Cystein carbamidomethylation and Methione oxidation as fixed and variable modifications respectively; mass values determined in monoisotopic mode with PMF experiments, the peptide charge state was set as +1 and the mass range was 70–4240*m*/*z*. For MS/MS experiments, the fragment mass tolerance was±0.3 Da and the mass range was 0–1100*m*/*z*. Searching was done against three version of UniprotKB/SwissProt database. Database A corresponds to version 57.1 (462764 sequences; 163773385 residues) released on April 2009 and database B corresponds to version 57.7 (497293 sequences; 175274722 residues) released on September 2009, database C corresponds to version 2014_05 (545388 sequences; 193948795 residues) released on May 2014.

### 1.6 Immunocytofluorescence

One thousand NA and TA cells were seeded as 50 µl drops on glass coverslips coated with 100 µg/ml polyornithine (Sigma) in MEM medium containing 10% FCS for 48 h. The cells were maintained in serum-free MEM medium for 24 h, rinsed twice in PBS, prior to be fixed with 4% paraformaldehyde.

The fixed cultures were washed 3 times in TBS buffer, permeabilized in 1% Triton-X100 (v/v) for 15 min and washed 3 times in TBS buffer. Non-specific binding was blocked using TBST containing non-fat milk powder (30 mg/ml) for 45 min. The cells were then incubated overnight at 4 °C with the following primary antibodies mixture diluted in blocking buffer: Anti-PK (Chemicon international, Ref: AB1235, Produced in goat,1/500), and Anti-glial fibrillary acidic protein (GFAP) (Dako, Ref: Z0334, produced in rabbit, 1/500). After 3 TBST washes, cells were incubated with FITC or RRX conjugated antibodies (Jackson Immunoresearch, Ref: 711-095-152 and 805-295-180, dilution 1:2500) for 1 h at RT and in the dark. Nucleus staining was achieved with 4′,6-diamidino-2-phenylindole (DAPI). The preparations were examined using Olympus confocal (Leica).

## Footnotes

Appendix A Supplementary data associated with this article can be found in the online version at 10.1016/j.dib.2014.09.004.
