# Supplementary material for: Data in support of metabolic reprogramming in transformed mouse cortical astrocytes: A proteomic study
Source: Data Brief. 2014 Nov 7;2:1–5. doi: 10.1016/j.dib.2014.09.004 (PMC4459766; doi:10.1016/j.dib.2014.09.004)
Supplement: Supplementary file 1 — Supplementary data [file mmc1.zip › Supplementary Figures and Table description.docx]

**Table 1.**

List of identified proteins whose abundance differs by at least 1.5-fold (p<0.05, student t-test) between normal astrocytes (NA) and transformed astrocytes (TA). Proteins have been classified into biological processes as defined in the Uniprot Database Keywords list. Spot ID is the unique identifier of each protein spot provided in the Decyder analysis. Protein ID is the item Entry name in the UniprotKB database. Average Ratio is the fold higher abundance in TA (positive values in green) or in NA (negative values in red). p-value is the Student t-test result for the comparison of abundance. Matched/Searched is the fraction of searched peptide masses that matched theoretical masses for the listed protein by PMF. Mowse score is the Mascot probability based scoring that the results are significant, either for PMF identification (threshold value = 59) or for MS/MS identification (threshold value = 42). % sequence coverage is the percent of the protein sequence that has been sequenced in the MS analysis. Number of matches is the number of non-overlapping identified peptides that resulted from the MS/MS search. Database: three version of SwissProt database were used, A corresponds to version 57.1 (462764 sequences; 163773385 residues, taxonomy: Mus musculus) released on April 2009, database B corresponds to version 57.7 (497293 sequences; 175274722 residues, taxonomy: Mus musculus) released on September 2009, database C corresponds to version 2014_05 (545388 sequences; 193948795 residues, taxonomy: Mammalia) released on May 2014.

**Figure 1.**
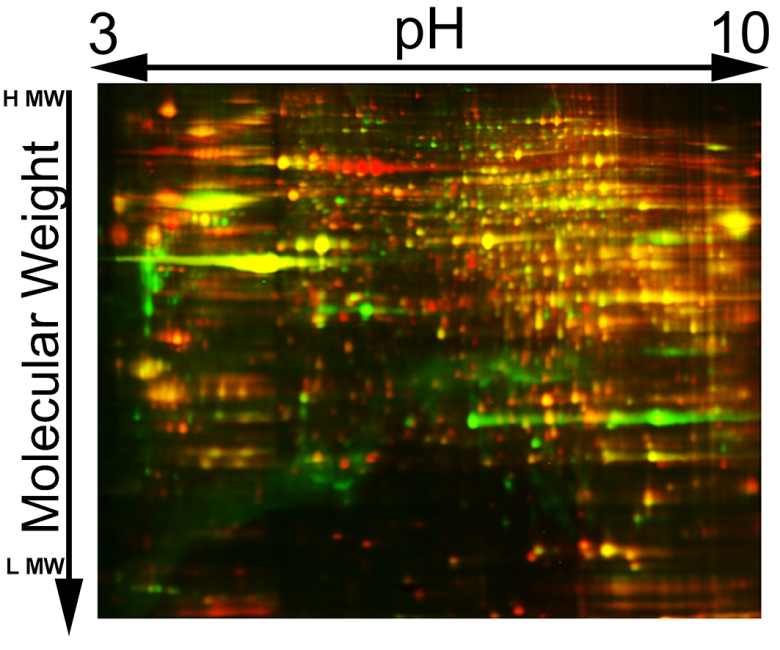
 Combined fluorescent image of a bidimensional separation of normal astrocytes (NA) (labeled with Cy3 and seen as green spots) and transformed astrocytes (TA) (labeled with Cy5 and seen as red spots) proteins. Twenty-five µg of each labeled extract were mixed together and subjected to bidimensional electrophoresis (focusing in a pH 3-10 range as first dimension and 12.5 % acrylamide SDS-PAGE (theoretical MW range: 12 – 180 kDa) as second dimension). The gel was then immediately scanned at the corresponding settings for Cy3 and Cy5 in a Typhoon 9400 laser-scanner. Contrast and luminosity have been adapted for appearance improvement.

**Figure 2.**

A) Graphical representations of the results of the 2D DIGE analysis of proteomes of NA and TA

B) Percentage of protein identification by mass spectrometry

**
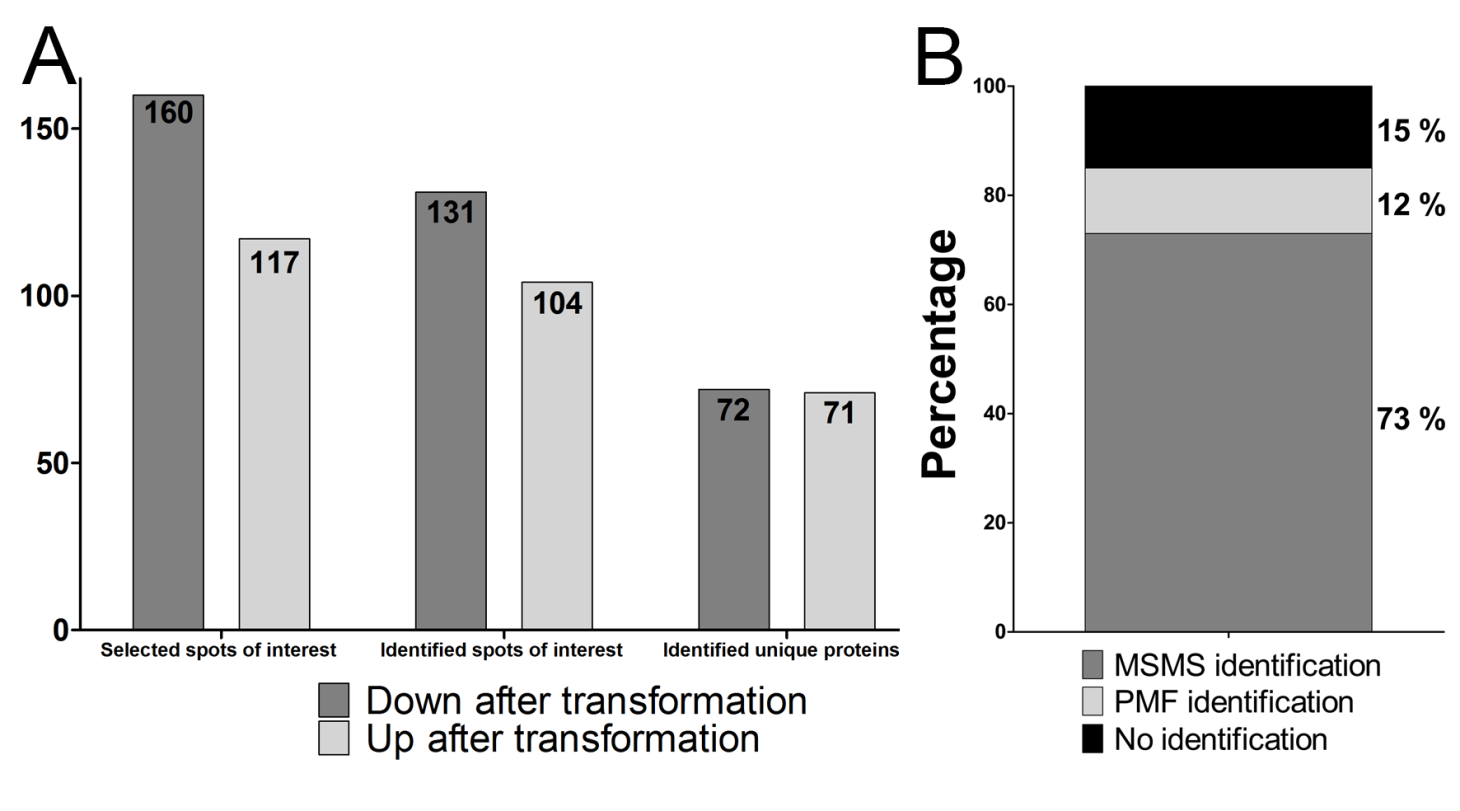
**

**Figure 3.**



Pyruvate kinase and GFAP immunostaining of NA and TA. The cells were immunostained with anti-PK (green) and anti-GFAP (red) antibodies. Nuclear staining (DAPI) is shown in blue. Scale bar represents 50 µm for all panels.

**Figure 4.**

Changes in the expression of alpha-enolase (ENO A) in NA and TA.

A) Graphical representation of the standardized abundance of three spots corresponding to ENO A that are more abundant in TA compared to NA,


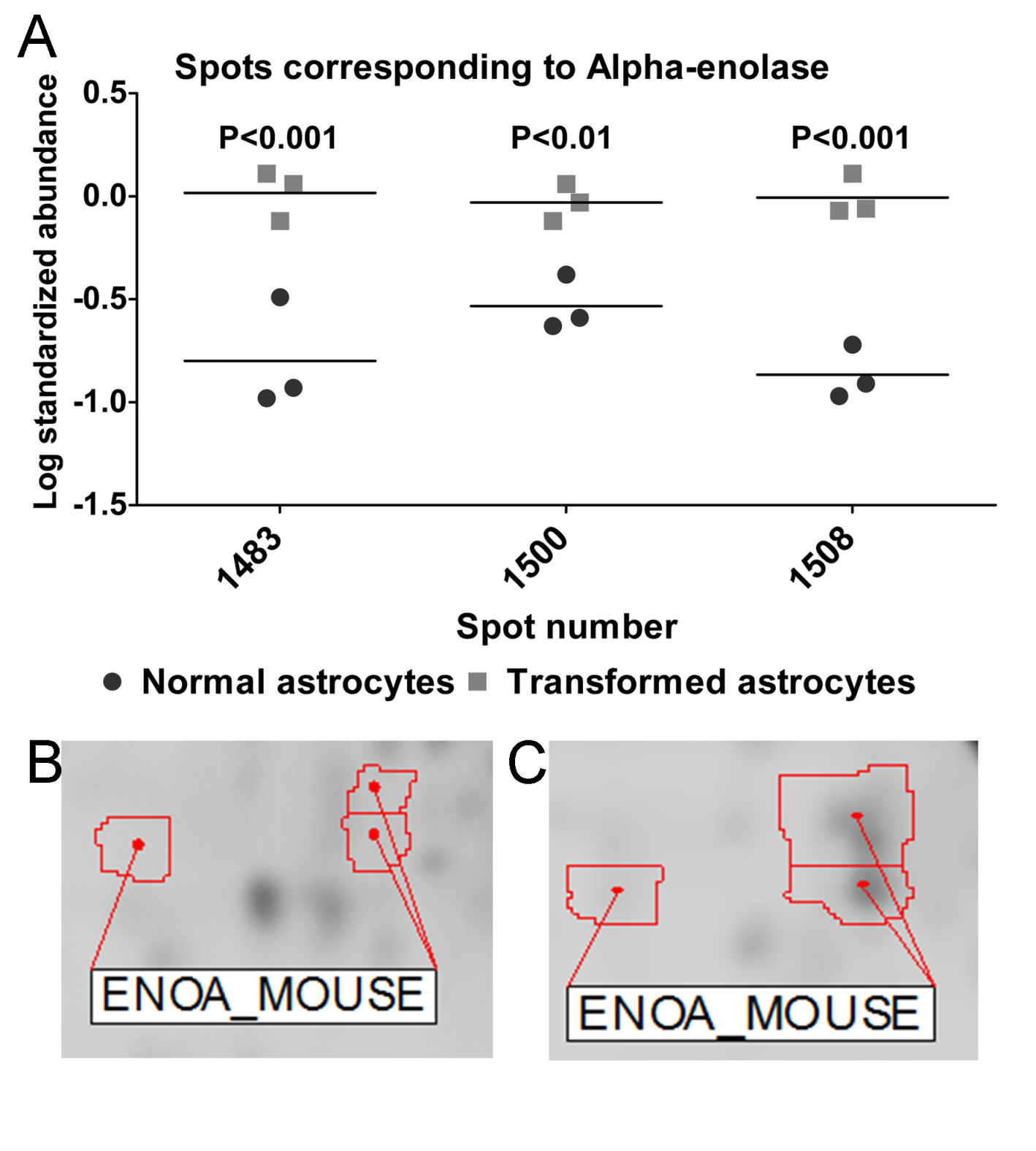
B, C) Distribution in bidimensional gels of Eno A spots in NA (B) and TA (C) protein extracts.

**Figure 5.**

Changes in the expression of pyruvate kinase M2 isoform (PKM 2) in NA and TA.

A) Graphical representation of the standardized abundance of seven spots corresponding to PKM 2 that are more abundant in TA compared to NA,

**
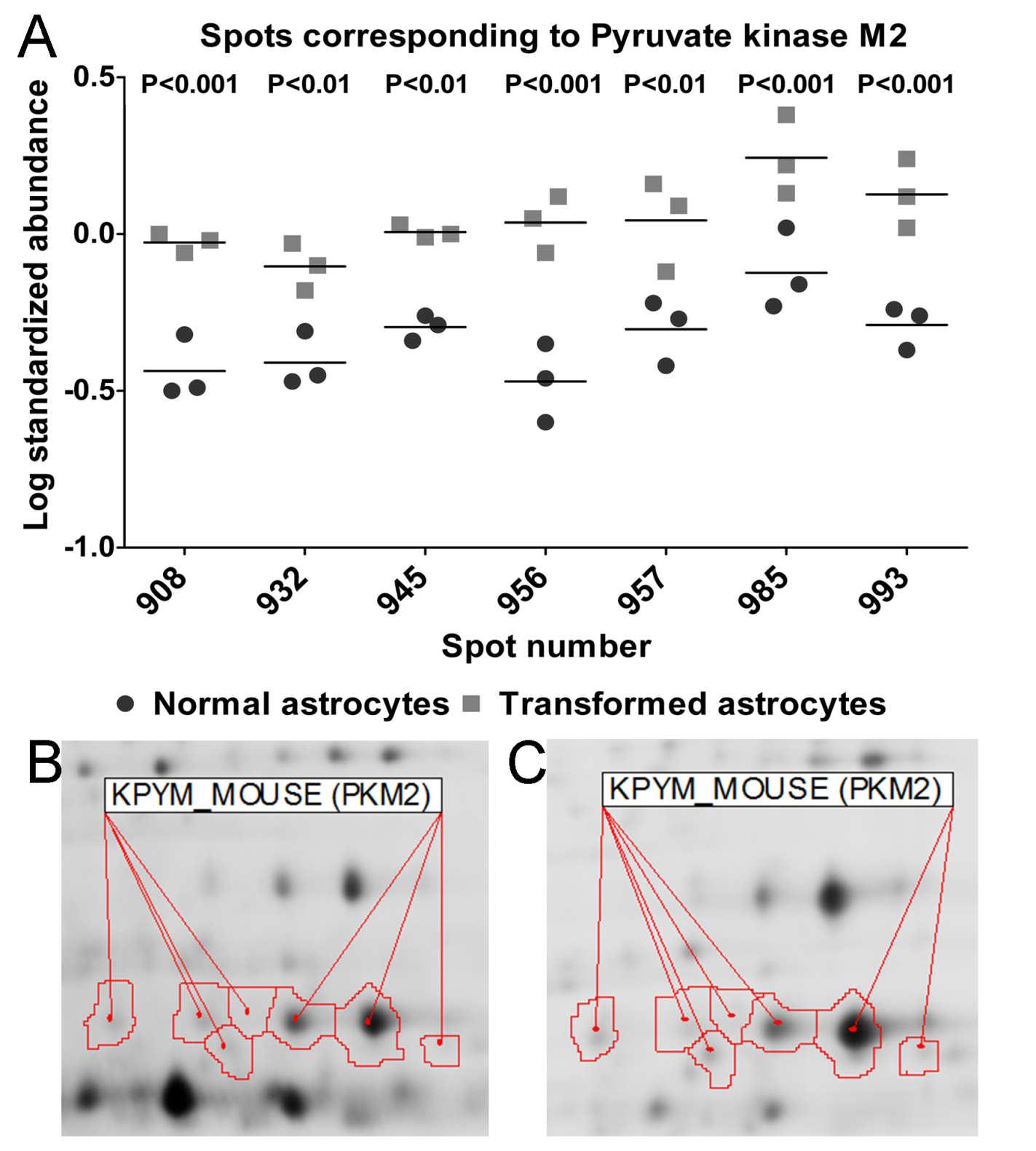
**B, C) Distribution in bidimensional gels of PKM 2 spots in NA (B) and TA (C) protein extracts.

**Figure 6.**

Changes in the expression of transaldolase (TALDO) in NA and TA.

A) Graphical representation of the standardized abundance of the spot corresponding to TALDO that is more abundant in TA compared to NA,


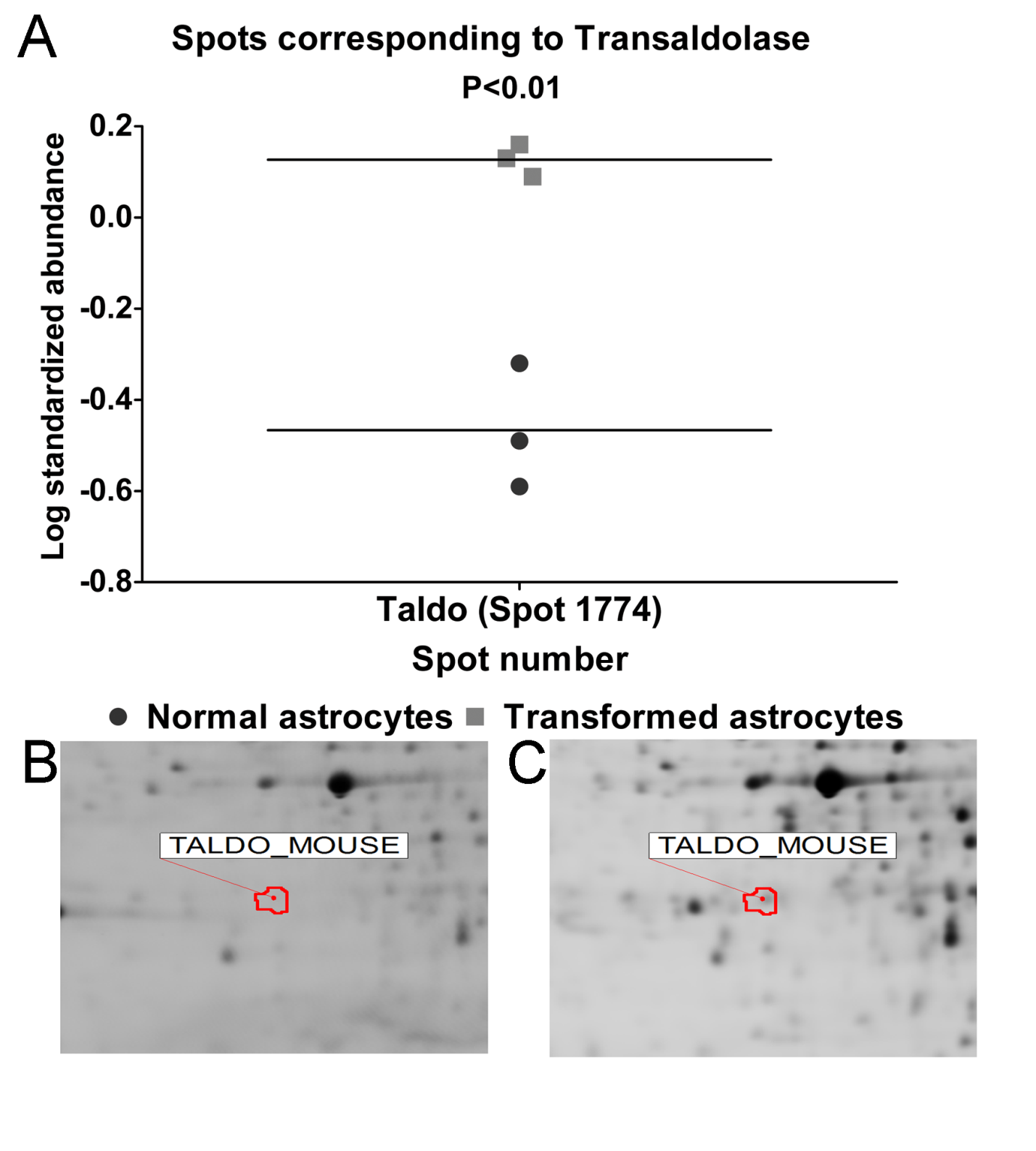
B, C) Distribution in bidimensional gels of TALDO spot in NA (B) and TA (C) protein extracts.

**Figure 7.**

Glyceraldehyd-3-phosphate dehydrogenase (G3P) as an example of an enzyme identified in several spots exhibiting opposite changes in abundance:

A) Graphical representation of the Log standardized abundances of four spots corresponding to G3P (1820, 1740, 1714 and 1766) showing that spots 1820,1714 and 1766 are more abundant in TA compared to NA, while spot 1740 shows a decrease in its relative abundance in TA.


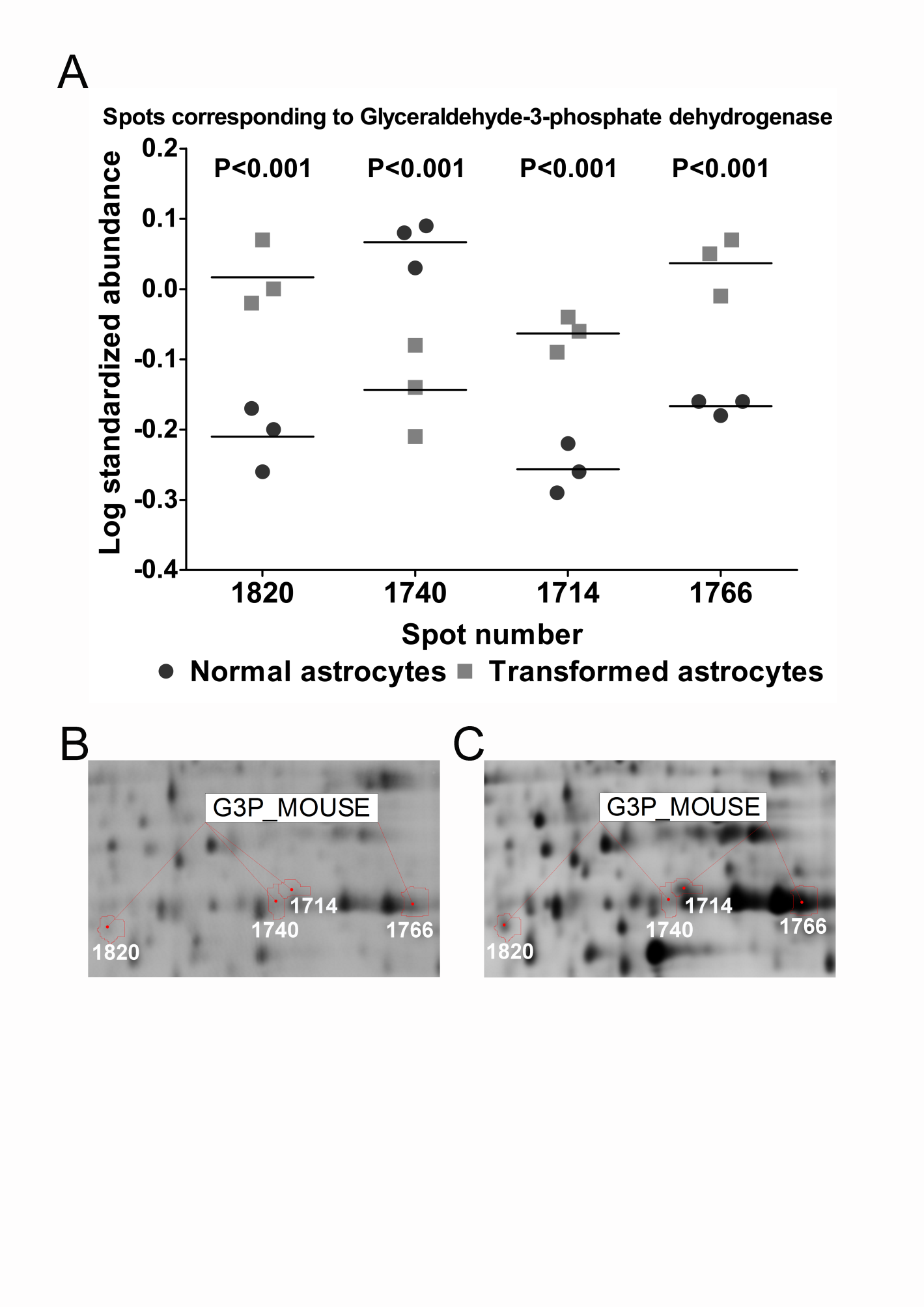
B, C) Distribution in bidimensional gels of identified G3P spots in NA (B) and TA (C) protein extracts.

**Figure 8.**

Changes in the expression of cytosolic and mitochondrial isoenzymes of malate dehydrogenase (respectively MDH C and MDH M) in NA and TA.

**
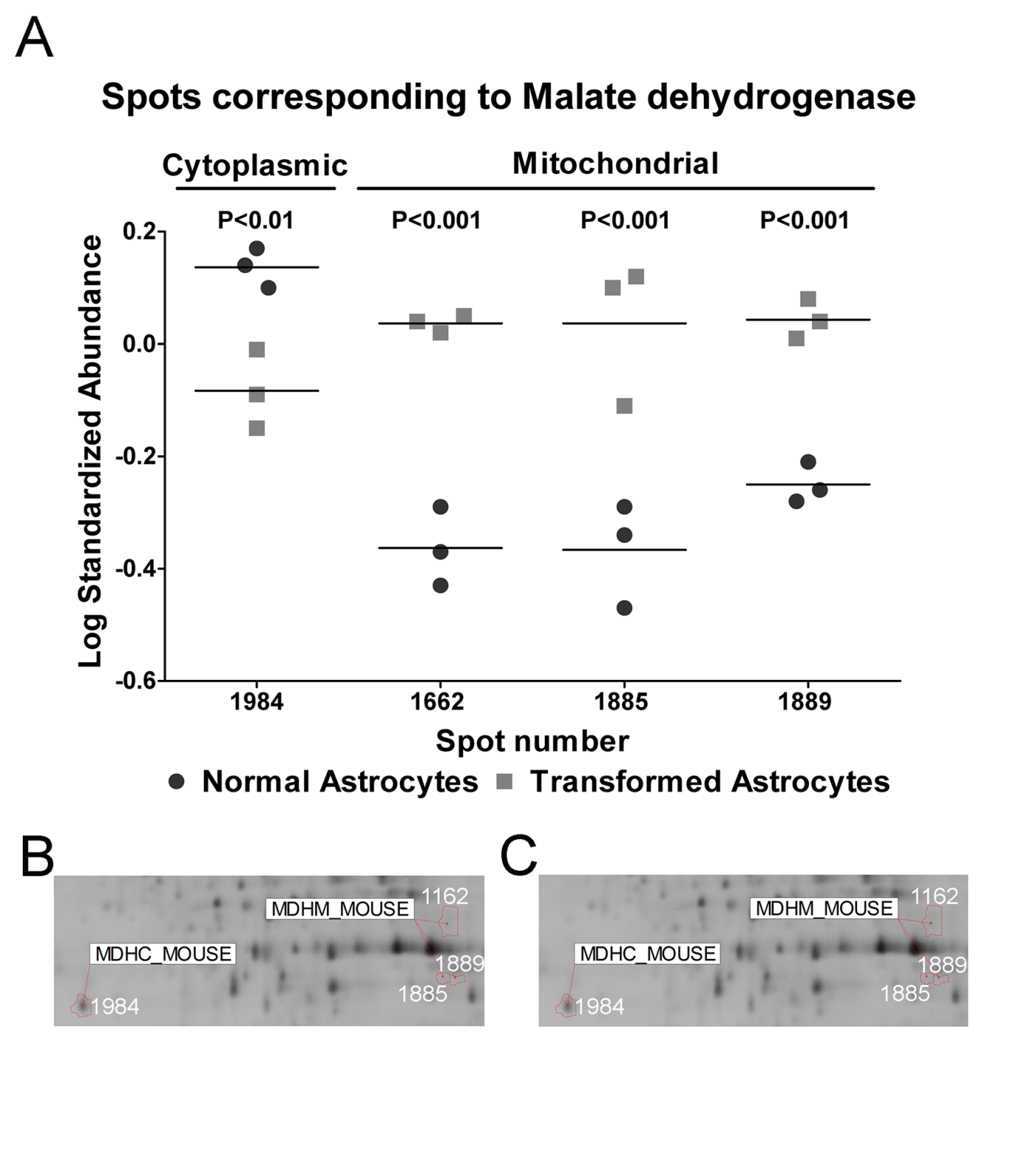
**A) Graphical representation of the standardized abundance of four spots corresponding to MDH showing that the spot corresponding to the cytosolic isoenzyme (MDH C, 1984) is less abundant in TA compared to NA, while the spots corresponding to mitochondrial isoenzymes (MDH M, 1162, 1885 and 1889) are more abundant in TA.B, C) Distribution in bidimensional gels of MDH spots in NA (B) and TA (C) protein extracts.

**Figure 9.**

Lactate dehydrogenase **(**LDH) isoenzymes A and B distribution in NA and TA.

A) Graphical representation of the Log standardized abundances of three spots corresponding to LDH A that are more abundant in TA compared to NA,

B) Graphical representation of the Log standardized abundances of four spots corresponding to LDH B that are less abundant in TA compared to NA,


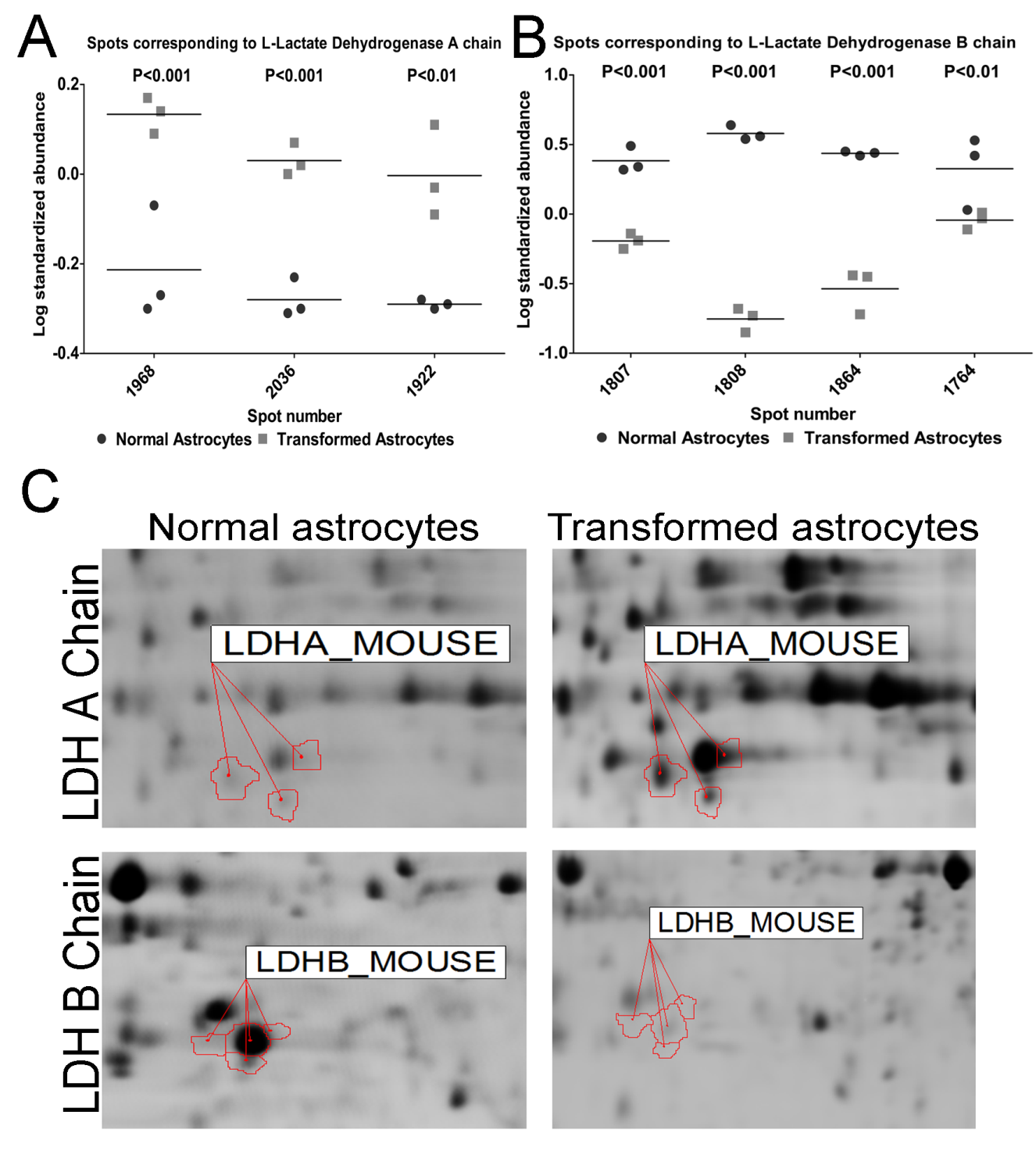
C) Distribution in bidimensional gels of identified LDH A and LDH B spots in NA and TA protein extracts.

**Figure 10.**

Changes in the expression of mitochondrial isocitrate dehydrogenase (IDHP) in NA and TA.

A) Graphical representation of the standardized abundance of two spots corresponding to IDHP that are less abundant in TA compared to NA,


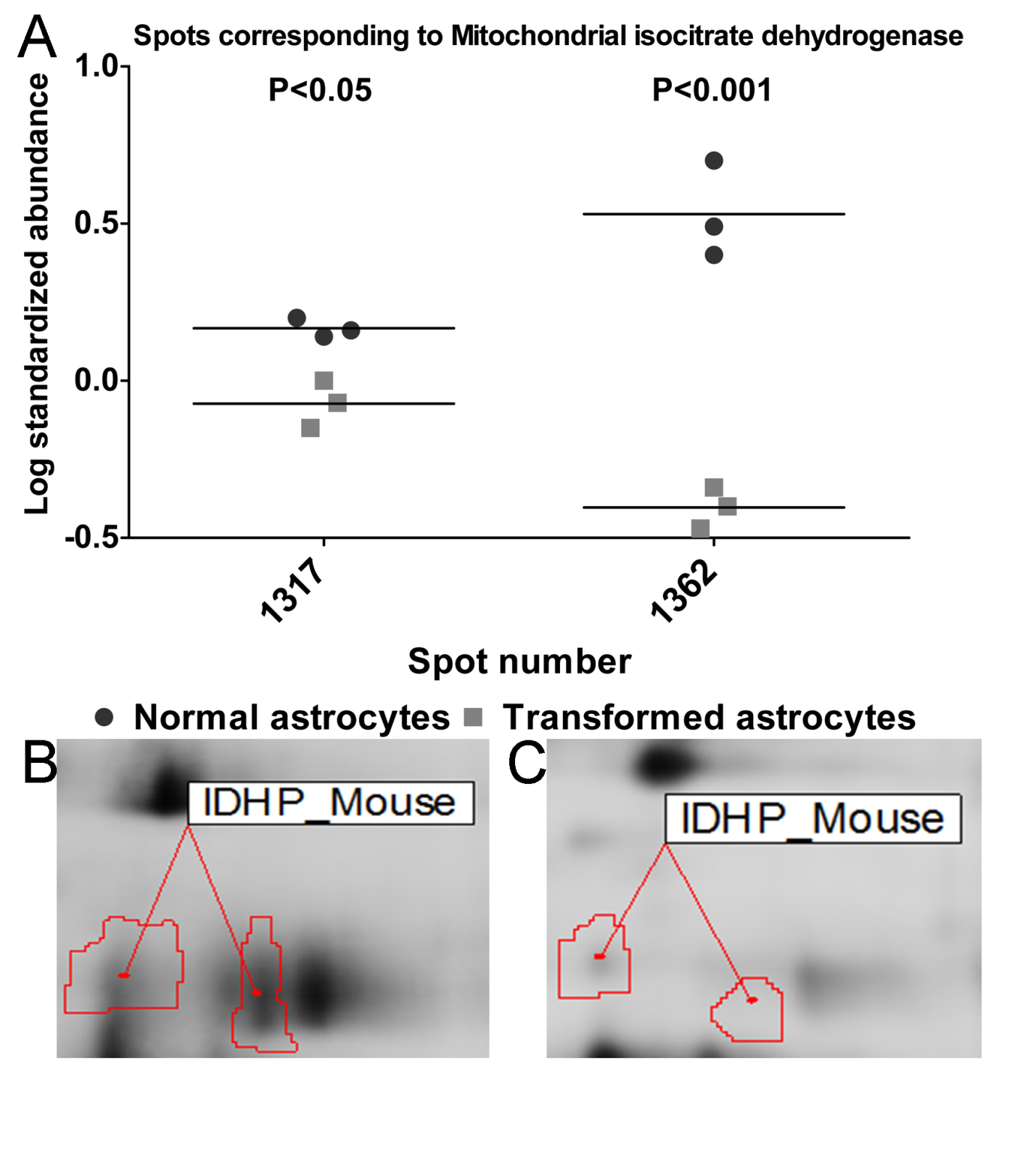
B, C) Distribution in bidimensional gels of IDHP spots in NA (B) and TA (C) protein extracts.

**Figure 11.**

Glutamate dehydrogenase (DHE 3) as an example of an enzyme identified in several spots and exhibiting opposite changes in abundance.

A) Graphical representation of the standardized abundance of six spots corresponding to DHE3 showing that spots 1168 and 1181 are more abundant in TA compared to NA, while spots 1076, 1073, 1071 and 1078 show a decrease in their relative abundance in TA.

**
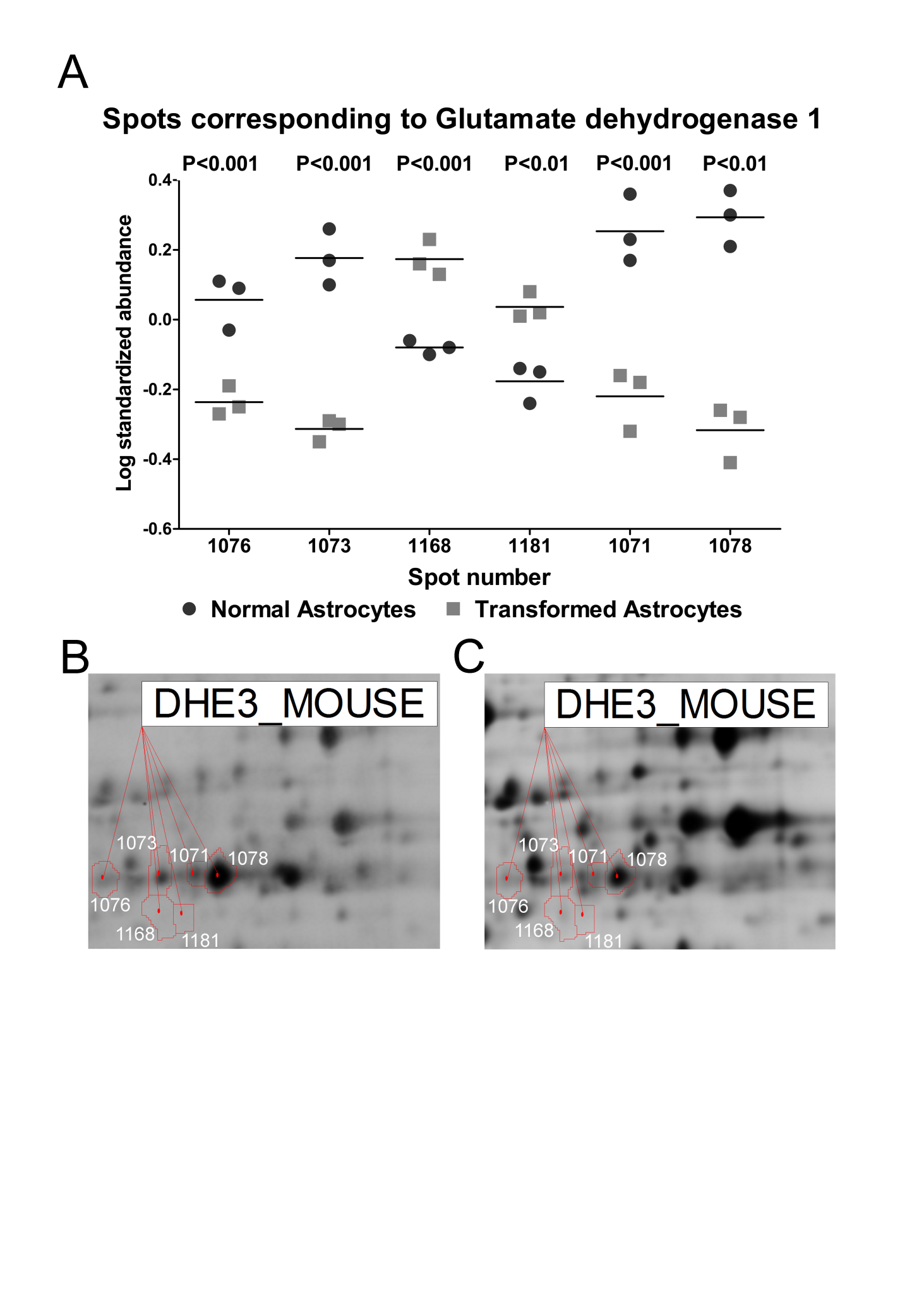
**B, C) Distribution in bidimensional gels of identified DHE3 spots in NA (B) and TA (C) protein extracts.

**Figure 12.**

Changes in the expression of glutamine synthetase (GS) in NA and TA.

A) Graphical representation of the standardized abundance of the spot corresponding to GS that is more less in TA compared to NA,


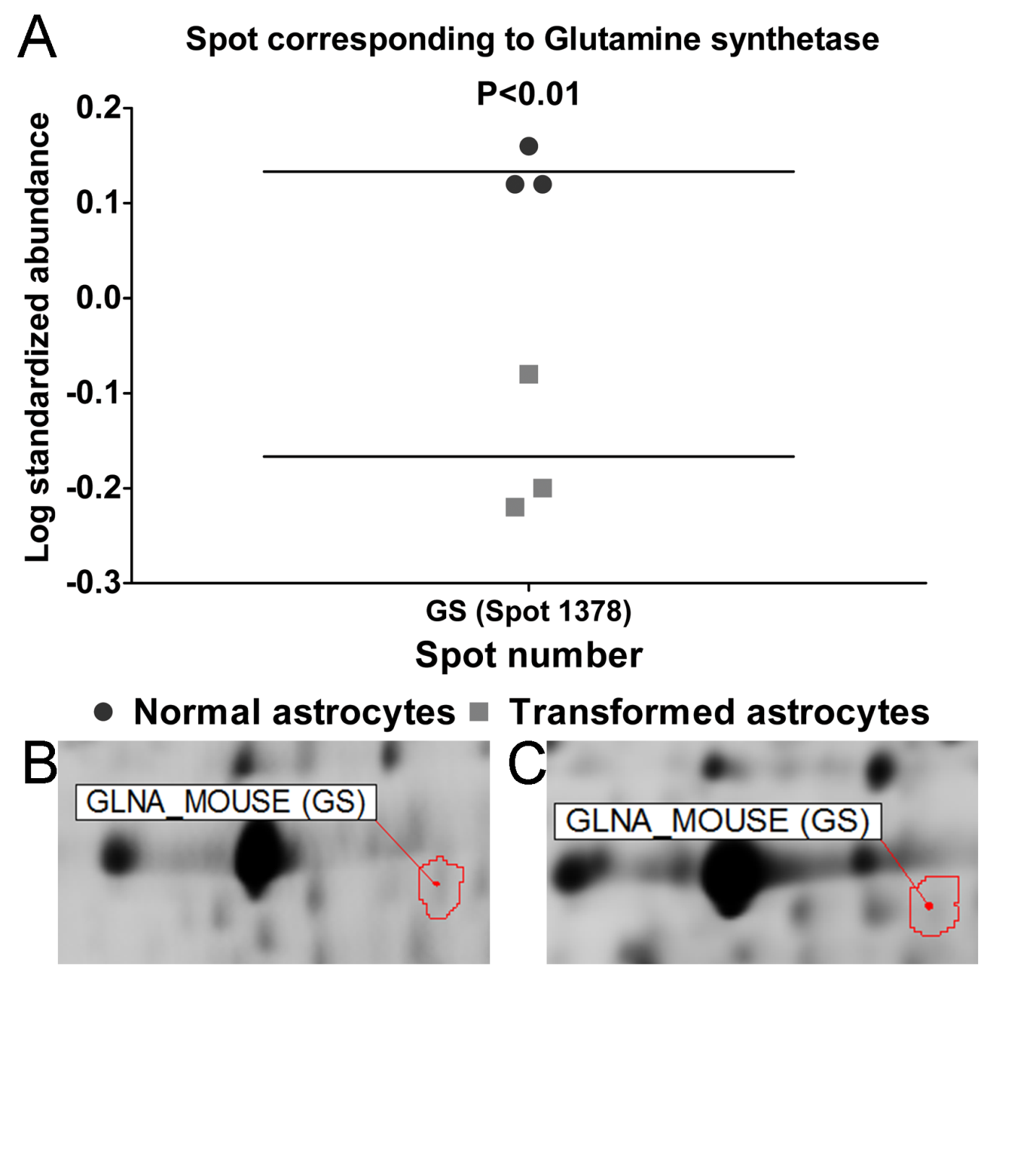
B, C) Distribution in bidimensional gels of GS spot in NA (B) and TA (C) protein extracts.

**Figure 13.**

Changes in the expression of peroxiredoxin-1 (PRDX 1) in NA and TA.

A) Graphical representation of the standardized abundance of two spots corresponding to PRDX 1 that are less abundant in TA compared to NA,


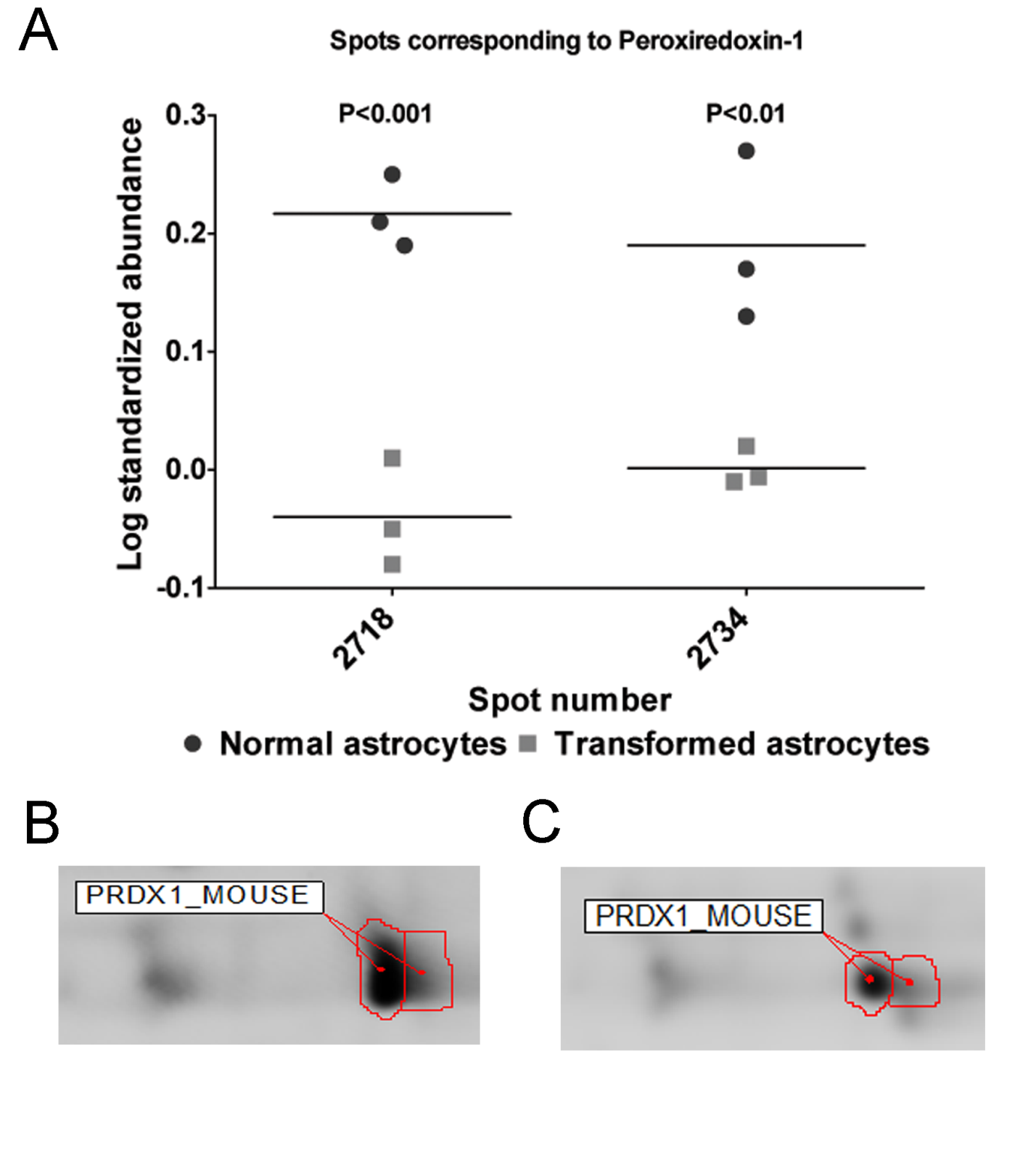
B, C) Distribution in bidimensional gels of PRDX 1 spots in NA (B) and TA (C) protein extracts.

**Figure 14.**

Changes in the expression of peroxiredoxin-6 (PRDX6) in NA and TA.

A) Graphical representation of the standardized abundance of six spots corresponding to PRDX 6 that are less abundant in TA compared to NA,


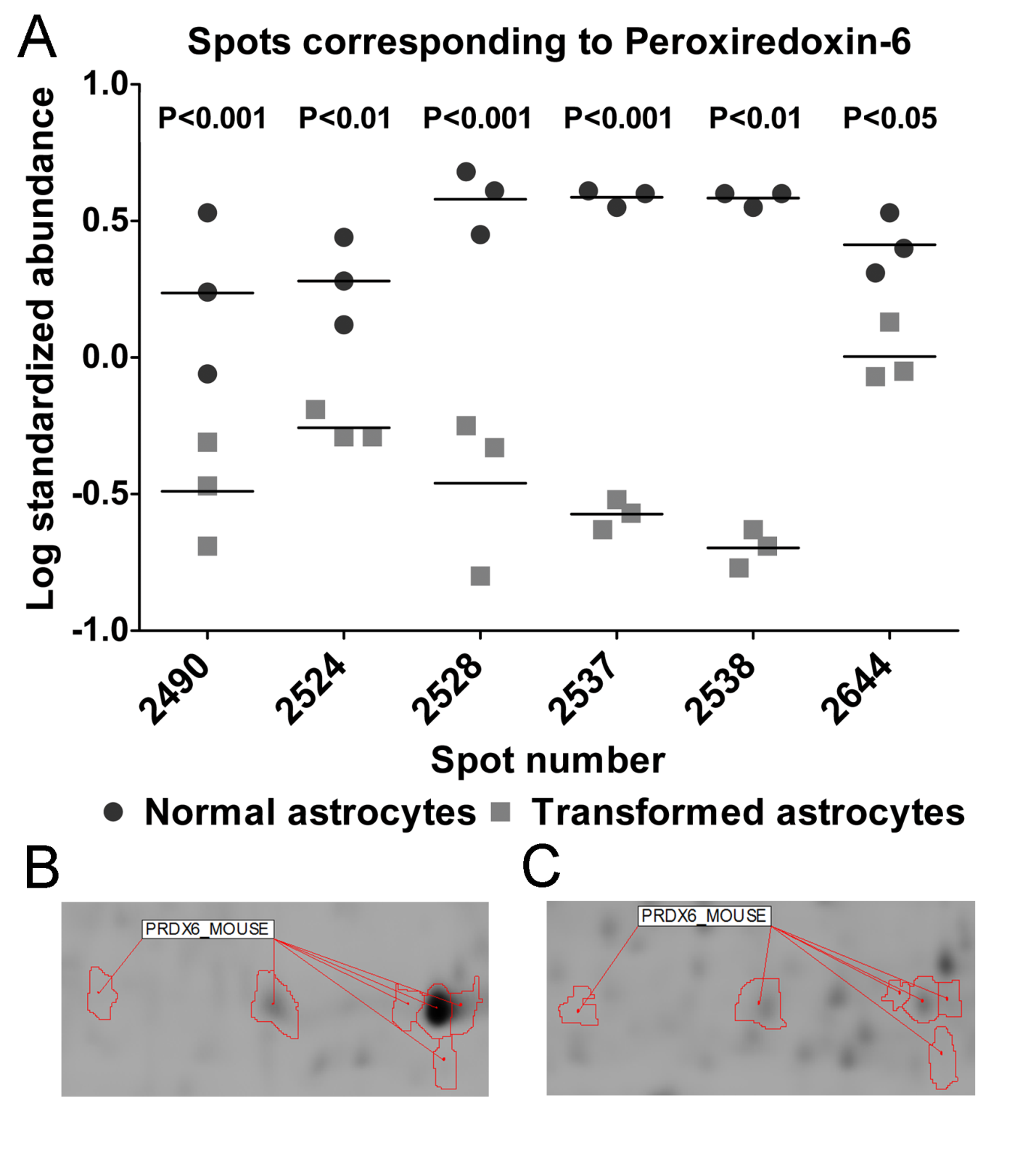
B, C) Distribution in bidimensional gels of PRDX 6 spots in NA (B) and TA (C) protein extracts.

**Figure 15.**

Changes in the expression of glutathione S- transferase M2, M5, A4 (respectively GSTM2, GSTM5 and GSTA4) in NA and TA.

A) Graphical representation of the standardized abundance of spots corresponding to GST M2, M5 and A4 that are less abundant in TA compared to NA,


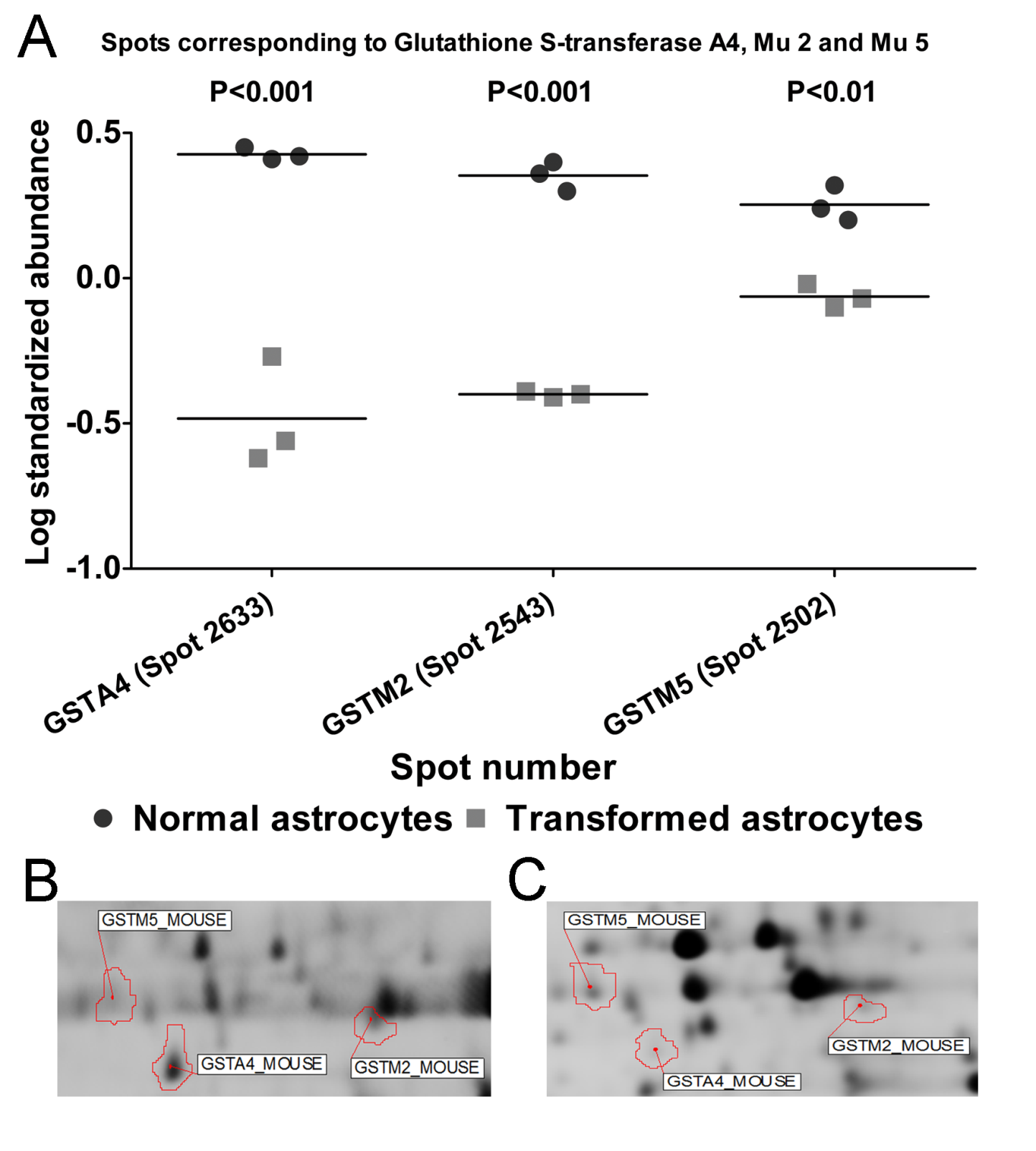
B, C) Distribution in bidimensional gels of GST M2, M5 and A4 spots in NA (B) and TA (C) protein extracts.

**Figure 16.**

Changes in the expression of glutathione S- transferase M1 (GSTM1) in NA and TA.

A) Graphical representation of the standardized abundance of fifteen spots corresponding to GST M1 that are less abundant in TA compared to NA,


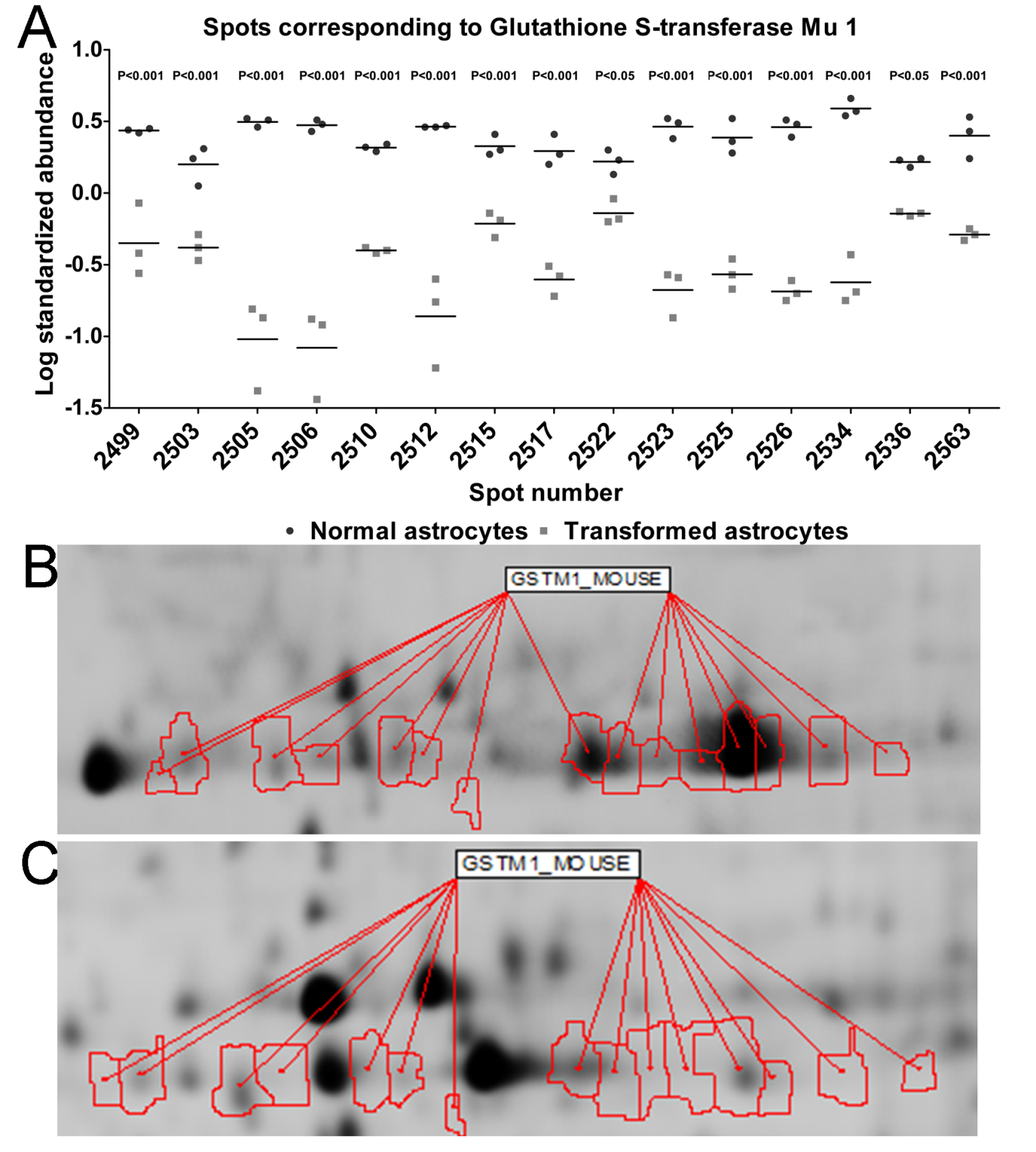
B, C) Distribution in bidimensional gels of GST M1 spots in NA (B) and TA (C) protein extracts.

**Figure 17.**

Changes in the expression of 78 kDa glucose-regulated protein (GRP 78) in NA and TA.

A) Graphical representation of the standardized abundance of six spots corresponding to GRP 78 that are more abundant in TA compared to NA,


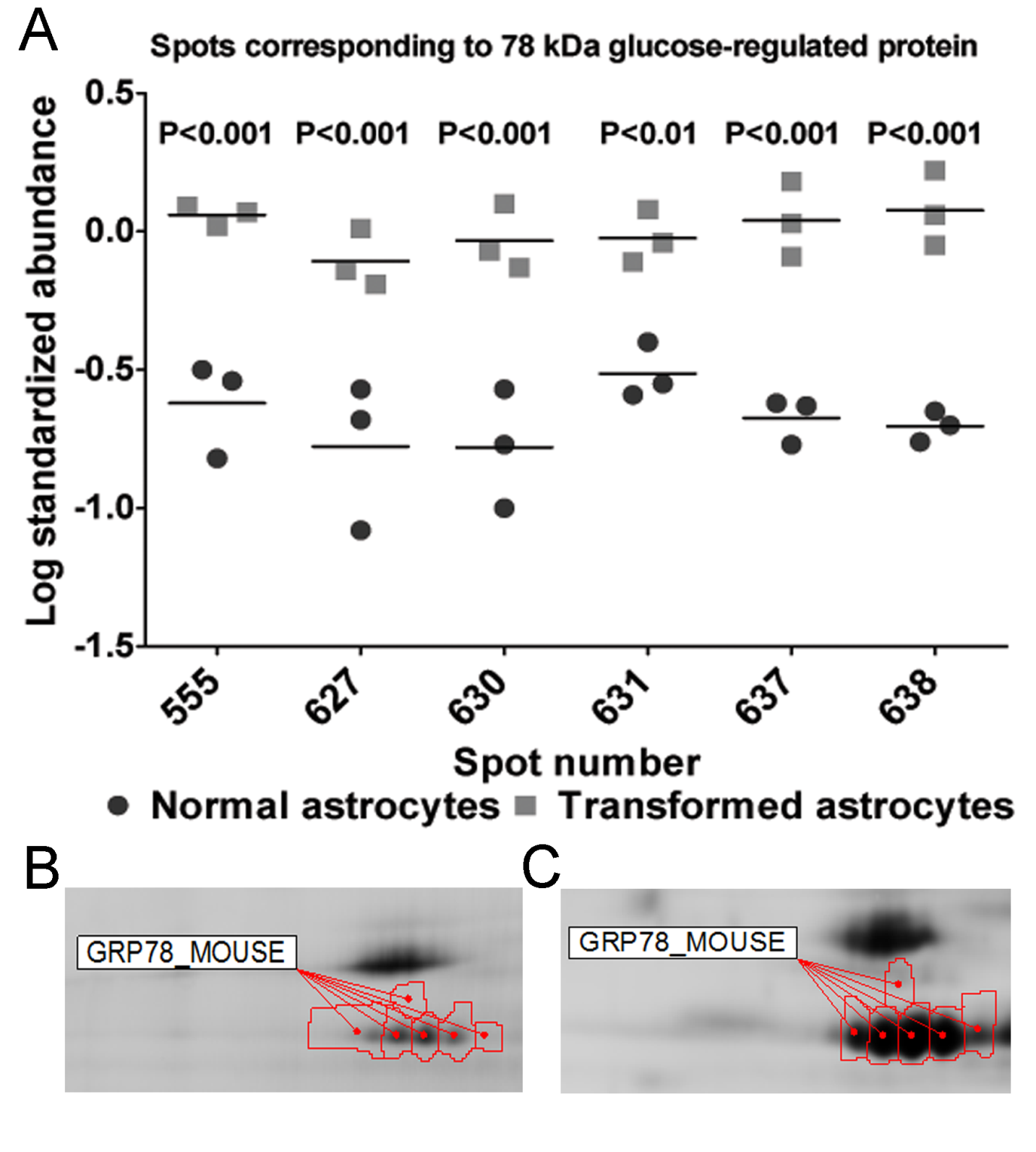
B, C) Distribution in bidimensional gels of GRP 78 spots in NA (B) and TA (C) protein extracts.
